# Postcholecystectomy Biliary Tract Traumatic Neuroma Mimicking Cystic Duct Stump Malignancy

**DOI:** 10.31486/toj.25.0073

**Published:** 2026

**Authors:** Ankit Rai, Subhash Chandra Soni, Meenakshi Rao, Binit Sureka, E Saikrishna, B Selvakumar, Peeyush Varshney, Lokesh Agarwal

**Affiliations:** ^1^ Department of Surgical Gastroenterology, All India Institute of Medical Sciences, Jodhpur, India; ^2^ Department of Pathology, All India Institute of Medical Sciences, Jodhpur, India; ^3^ Department of Radiology, All India Institute of Medical Sciences, Jodhpur, India

**Keywords:** *Biliary tract*, *biliary tract neoplasms*, *cholangiocarcinoma*, *cystic duct*, *diagnostic error*, *neuroma*

## Abstract

**Background:**

Biliary tract traumatic neuroma is a rare benign hyperplasia of nerve tissue that follows some form of trauma. These lesions closely mimic malignancy, and a conclusive diagnosis is difficult to establish.

**Case Report:**

We report the case of a 65-year-old female who presented with jaundice with cholestatic features, abdominal pain, and intermittent episodes of fever. She had undergone open cholecystectomy 3 years prior for gallstone disease. Her liver function tests showed direct hyperbilirubinemia with elevated alkaline phosphatase. Serum CA 19-9 was >3,000 U/L. Cross-sectional imaging showed a nodular mass at the hilum causing extrinsic compression of the common hepatic duct with resultant bilobar intrahepatic biliary dilatation. Endoscopic ultrasound showed an eccentric thickening of the common hepatic duct, just below the hilum. Malignant common hepatic duct stricture was provisionally diagnosed, and the patient underwent open extrahepatic biliary excision with Roux-en-Y hepaticojejunostomy. The proximal and distal resection margins were confirmed negative for dysplasia or malignancy on frozen section. Final histopathologic examination showed conglomeration of nerve fascicles and Schwann cells in a fibrotic stroma, features suggestive of a traumatic neuroma.

**Conclusion:**

Traumatic neuroma of the biliary tree is a rare entity and mimics biliary tract malignancy. Preoperative diagnosis of traumatic neuromas is usually difficult, and surgery is indicated in cases of diagnostic uncertainty.

## INTRODUCTION

Traumatic or amputation neuroma, a nonneoplastic proliferation of nerve tissue, is a type of reactive hyperplasia that develops at the transected end of a nerve following primary or surgical trauma and is characterized by fibrosis and disorganized growth of nerve fascicles.^1^ Traumatic neuromas are most commonly encountered following nerve injury associated with limb amputation, orthopedic procedures, and head and neck surgeries and represent an attempt at self-repair that results in formation of an entangled mass of Schwann cells, fibroblasts, and axons within a fibrotic stroma.^1,2^

Biliary tract traumatic neuromas are relatively rare benign lesions that are often identified incidentally on histopathologic examination.^3^ Because published data are limited to case reports and small series, the true incidence and any potential sex predilection remain undefined. Reported latency between biliary surgery and diagnosis varies widely, ranging from months to several years.^4^ Biliary tract traumatic neuromas may remain clinically silent or present with nonspecific symptoms such as abdominal pain, cholestatic liver function abnormalities, obstructive jaundice, and even acute cholangitis.^4^ Because of these nonspecific features and imaging findings, such lesions may be mistaken for postcholecystectomy syndrome, choledocholithiasis, or even biliary tract malignancy.^5,6^ We report a rare case of postcholecystectomy traumatic neuroma mimicking mid common bile duct malignancy.

## CASE REPORT

A 65-year-old female presented with progressively increasing yellowish discoloration of her eyes and urine, acholic stools, and generalized pruritus for the prior 4 months, as well as right upper abdominal pain with fever and chills for the prior 2 months. She had no history of gastrointestinal bleed, anorexia, or weight loss. The patient had undergone open cholecystectomy 3 years prior for symptomatic gallstone disease. She had no postprocedural complications; histopathology showed features of chronic cholecystitis with cholelithiasis.

The patient's total leukocyte count was 12,670 cells/mm^3^ (reference range, 4,000-11,000 cells/mm^3^), and liver function tests showed a cholestatic pattern with elevated serum bilirubin of 3.33 mg/dL (reference range, 0.2-1.2 mg/dL), direct bilirubin of 2.24 mg/dL (reference range, 0-0.3 mg/dL), and alkaline phosphatase of 1,584 U/L (reference range, 40-129 U/L). Mild transaminitis was also noted; aspartate aminotransferase was 87 U/L (reference range, ≤40 U/L) and alanine aminotransferase was 175 U/L (reference range, ≤40 U/L). Tumor markers were also elevated; serum CA 19-9 and carcinoembryonic antigen were 3,333 U/L (reference range, ≤37 U/L) and 3.639 U/L (reference range, ≤5.0 U/L), respectively.

**Figure 1.**
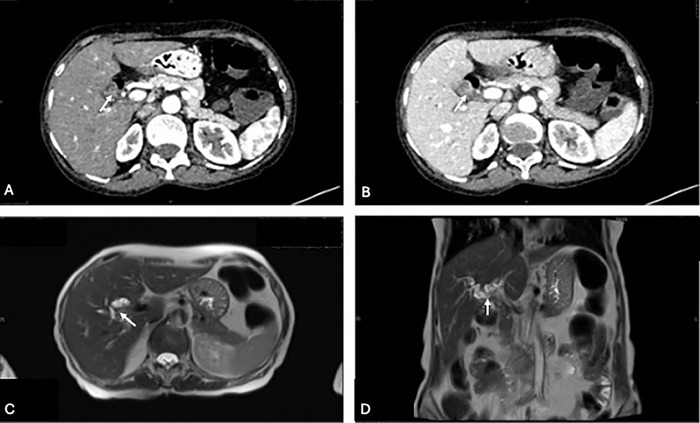
(A, B) Contrast-enhanced computed tomography of the abdomen shows a focal nodular enhancing lesion (arrows) involving the proximal common hepatic duct. (C) Axial T2-weighted magnetic resonance image shows an ill-defined isointense to hypointense lesion (arrow) at the proximal common hepatic duct. (D) Magnetic resonance cholangiopancreatography image shows focal narrowing of the proximal common hepatic duct (arrow) with upstream dilatation of the right hepatic duct, left hepatic duct, and intrahepatic biliary radicles, raising suspicion for a Bismuth-Corlette type I hilar cholangiocarcinoma.

**Figure 2.**
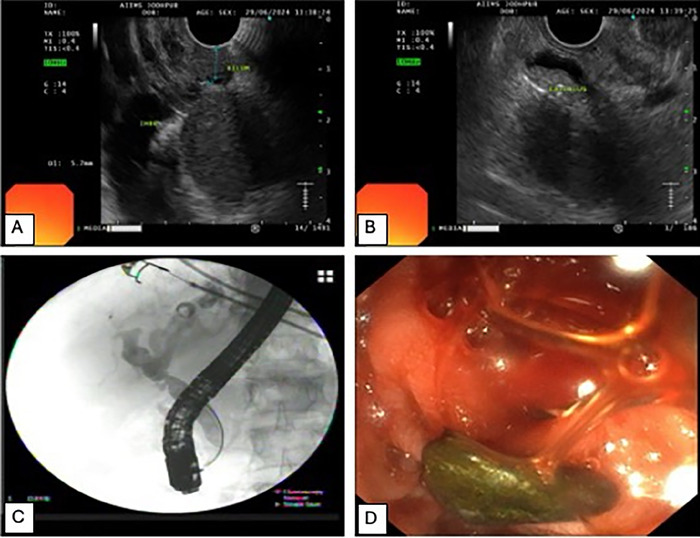
(A, B) Endoscopic ultrasound images show eccentric thickening of the proximal common hepatic duct with upstream ductal dilatation and distal common bile duct calculi. (C) Endoscopic retrograde cholangiopancreatography image shows a dilated common bile duct with intraluminal filling defects consistent with choledocholithiasis. (D) Endoscopic view shows extraction of a bile duct calculus during endoscopic retrograde cholangiopancreatography.

Contrast-enhanced computed tomography of the abdomen showed a 14 × 11-mm nodular enhancing lesion just below the primary confluence, causing moderate bilobar intrahepatic biliary dilatation with intraductal stones (Figures 1A and 1B). The thickening extended along the common hepatic duct, causing short segment stricture. The distal common bile duct was dilated with multiple calculi. T2-weighted magnetic resonance imaging showed a 14 × 15-mm ill-defined isointense to hypointense lesion at the proximal common hepatic duct (Figure 1C). Magnetic resonance cholangiopancreatography revealed narrowing and dilatation of the proximal right hepatic duct, left hepatic duct, and intrahepatic biliary radicles. Filling defects were also noted, with imaging findings suggestive of a Bismuth-Corlette type I hilar cholangiocarcinoma (Figure 1D). Endoscopic ultrasound showed an eccentric thickening of the common hepatic duct up to 5 mm, just below the hilum; however, no mass lesion was seen (Figures 2A and 2B). The lower common bile duct was dilated (7 mm) with multiple calculi. Endoscopic retrograde cholangiopancreatography showed a dilated common bile duct with a focal narrowing in the proximal duct and intraluminal filling defects consistent with choledocholithiasis (Figure 2C). Stone extraction was performed, followed by biliary stenting in view of low-grade cholangitis (Figure 2D).

The provisional diagnosis was cystic duct stump malignancy extending into the common hepatic duct or mid common bile duct cholangiocarcinoma, and the patient underwent open extrahepatic bile duct excision with Roux-en-Y hepaticojejunostomy. Intraoperatively, a 20 × 20-mm firm mass was discovered at the cystic duct stump infiltrating into the common hepatic duct. Extrahepatic biliary tree excision was performed proximally to the base of the hilum and distally to the retropancreatic common bile duct. Frozen sections from both the proximal and distal margins of the resection were free of malignancy. Regional lymph nodal clearance was also performed.

**Figure 3.**
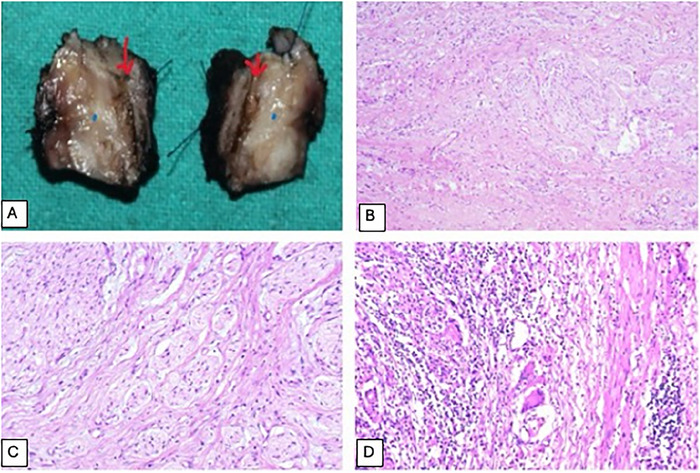
(A) Photograph of the excised bile duct segment shows a firm, gray-white nodular lesion at the cystic duct stump/common hepatic duct region (arrows). (B) Photomicrograph (hematoxylin and eosin [H&E] stain, low-powered magnification) shows disorganized proliferation of nerve fascicles embedded within a collagenized to fibrotic stroma. (C) Photomicrograph (H&E stain, high-powered magnification) shows haphazardly arranged mature nerve bundles composed of axons and Schwann cells, without cytologic atypia or mitotic activity. (D) Photomicrograph (H&E stain) shows associated chronic inflammatory infiltrate and fibrosis with no evidence of malignancy.

The histopathologic examination showed a haphazard and disordered proliferation of nerve fascicles and perineural cells in a collagenized to fibrotic stroma, as well as many preserved nerve bundles with intact endoneurium, perineurium, and epineurium along with small, rejuvenated nerve fibers. Moderate to focal dense chronic inflammation in the wall of the bile duct predominantly comprised lymphocytes and a few histiocytes, focal foreign body (suture material), and surrounding foreign body giant cell reaction (Figure 3). The lymph nodes showed reactive lymphoid hyperplasia. Overall, the histologic features indicated traumatic neuroma without any evidence of intraepithelial neoplasia or invasive malignancy.

The patient's postoperative stay was uneventful, and she was discharged on postoperative day 4. At 1-year follow-up, the patient was doing well without any symptoms.

## DISCUSSION

In the biliary tract, traumatic neuromas arise from disorganized regeneration of periductal nerve fibers after surgical or inflammatory insult, most commonly following cholecystectomy, bile duct exploration, or biliary reconstruction. Because these lesions are benign and often small, they may remain clinically unrecognized.^4^ Biliary tract traumatic neuromas are often diagnosed in middle-aged to older adults and may be detected incidentally on histopathologic examination following biliary surgery or may present with nonspecific symptoms.^1,3,5,7-12^

Preoperative diagnosis of biliary tract traumatic neuroma is challenging. Cross-sectional imaging frequently demonstrates focal biliary strictures or mass-like lesions that are difficult to distinguish from cholangiocarcinoma or benign biliary strictures.^4^ Tsitouridis et al reported that the signal intensity of nerve tissue is the same as soft tissue and therefore nonspecific.^10^ Magnetic resonance cholangiopancreatography may demonstrate focal ductal narrowing with or without upstream biliary dilatation, while endoscopic evaluation often reveals a localized stricture.^13^

Martinsone et al described a case of traumatic bile duct neuroma presenting more than a decade after biliary injury that was radiologically suspected as hilar cholangiocarcinoma, with definitive diagnosis made only on postoperative histopathology.^5^ Cao et al reported obstructive jaundice caused by a bile duct traumatic neuroma, emphasizing the difficulty of preoperative differentiation from malignancy and the importance of intraoperative pathologic assessment.^11^ Intraoperative frozen section examination may serve as a useful adjunct to confirm benign pathology, exclude malignancy, and guide the extent of bile duct resection and biliary reconstruction.^12^

Because of their rarity, indolent course, and nonspecific imaging features, biliary tract traumatic neuromas are often misinterpreted preoperatively as biliary tract malignancies, with the correct diagnosis established only after surgical excision and histopathologic evaluation.^1,3,5,7-12^ Histopathologic examination remains the gold standard for diagnosis of biliary tract traumatic neuroma. As noted previously, these lesions are characterized by a haphazard proliferation of mature nerve fascicles composed of axons, Schwann cells, and fibroblasts embedded within a collagenized or fibrotic stroma, with absence of cytologic atypia, mitotic activity, or invasive growth.^1,2,4,14^ Associated chronic inflammation and foreign body–type reaction may be present, particularly in postsurgical patients, supporting a reactive rather than neoplastic process. Immunohistochemical staining with neural markers such as S-100 protein can aid in confirming the diagnosis in equivocal cases.^4^

Management of biliary tract traumatic neuroma depends on symptomatology and diagnostic certainty. Asymptomatic lesions with a confident preoperative diagnosis may be managed conservatively.^4^ However, in symptomatic patients or when malignancy cannot be excluded, surgery is the definitive treatment when intervention is required.^14^ Traumatic neuromas are insensitive to chemotherapy and radiotherapy.^14^ The potential morbidity, including long-term complications associated with surgical intervention, must be considered before surgical planning. Prognosis following complete excision is excellent, and recurrence is rare.^4^

## CONCLUSION

Traumatic neuroma of the biliary tree is a rare benign entity that can closely mimic biliary tract malignancy in clinical and radiologic presentation. This case highlights the diagnostic challenge posed by biliary tract traumatic neuroma in a patient with prior cholecystectomy who presented with obstructive jaundice and elevated tumor markers that closely mimicked hilar cholangiocarcinoma, underscores the importance of considering biliary tract traumatic neuroma in the differential diagnosis of postcholecystectomy biliary strictures, and reinforces the critical role of histopathologic evaluation in establishing the final diagnosis.

## Notes

*This article meets the Accreditation Council for Graduate Medical Education and the American Board of Medical Specialties Maintenance of Certification competencies for Patient Care and Medical Knowledge.*
